# Simultaneous trimodal PET-MR-EEG imaging: Do EEG caps generate artefacts in PET images?

**DOI:** 10.1371/journal.pone.0184743

**Published:** 2017-09-13

**Authors:** Ravichandran Rajkumar, Elena Rota Kops, Jörg Mauler, Lutz Tellmann, Christoph Lerche, Hans Herzog, N. Jon Shah, Irene Neuner

**Affiliations:** 1 Institute of Neuroscience and Medicine 4 (INM4), Forschungszentrum Juelich, Juelich, Germany; 2 Department of Psychiatry, Psychotherapy and Psychosomatics, RWTH Aachen University, Aachen, Germany; 3 JARA – BRAIN – Translational Medicine, Juelich, Germany; 4 Department of Neurology, RWTH Aachen University, Aachen, Germany; 5 Department of Electrical and Computer Systems Engineering, and Monash Biomedical Imaging, School of Psychological Sciences, Monash University, Melbourne, Victoria, Australia; Banner Alzheimer's Institute, UNITED STATES

## Abstract

Trimodal simultaneous acquisition of positron emission tomography (PET), magnetic resonance imaging (MRI), and electroencephalography (EEG) has become feasible due to the development of hybrid PET-MR scanners. To capture the temporal dynamics of neuronal activation on a millisecond-by-millisecond basis, an EEG system is appended to the quantitative high resolution PET-MR imaging modality already established in our institute. One of the major difficulties associated with the development of simultaneous trimodal acquisition is that the components traditionally used in each modality can cause interferences in its counterpart. The mutual interferences of MRI components and PET components on PET and MR images, and the influence of EEG electrodes on functional MRI images have been studied and reported on. Building on this, this study aims to investigate the influence of the EEG cap on the quality and quantification of PET images acquired during simultaneous PET-MR measurements. A preliminary transmission scan study on the ECAT HR+ scanner, using an Iida phantom, showed visible attenuation effect due to the EEG cap. The BrainPET-MR emission images of the Iida phantom with [^18^F]Fluordeoxyglucose, as well as of human subjects with the EEG cap, did not show significant effects of the EEG cap, even though the applied attenuation correction did not take into account the attenuation of the EEG cap itself.

## Introduction

The modalities of positron emission tomography (PET), magnetic resonance imaging (MRI), and electroencephalography (EEG) were combined into a single trimodal approach in order to best utilise their complementary features. The trimodal imaging set-up yields high spatial resolution MR images, high temporal resolution EEG signals, and metabolic PET images simultaneously in a single scanning session [1]. The feasibility of measuring PET-MR-EEG data simultaneously in single session has already been reported [2–4] and the benefits of such a set-up are numerous. For example, the use of a PET-MR-EEG set-up can advance the understanding of brain structure and function and can be used to identify new biomarkers for neurological and psychiatric disorders, as well as improving accuracy in early diagnosis [5]. A potential *in vivo* marker for neocortical neuronal loss in Alzheimer’s disease was found based on evidence from PET, MRI, and EEG [6]. Furthermore, this setup is especially desirable for pharmacological challenge studies where a sequential design would add confounding factors [7]. A PET-MR-EEG set-up could also be used to improve pre-surgical evaluation and post-surgical monitoring in epilepsy patients in order to identify and localise the epileptogenic zone more quickly [8,9]. Further possibilities relating to the potential application of multimodal neuroimaging are well reviewed and discussed in the literature [10–12]. Interpretations and quantitative results from trimodal studies should, however, consider mutual interferences between the three modalities. In the literature, studies on the mutual interferences of PET and MR components in hybrid PET-MR scanners on MR and PET images [13–16], on the successful removal of MR-related artefacts from EEG signals [17–19], as well as on the influence of EEG electrodes on functional MRI (fMRI) blood oxygenation level dependent (BOLD) signal [20] have already been reported on. Simultaneous PET-EEG measurements were performed by a number of groups using PET only scanners [21–24] and applying 68Ge-transmission based attenuation correction. It is not necessary to investigate and analyse the attenuation effect of an EEG cap while using PET only scanners for simultaneous PET-EEG measurements because the attenuation map generated during transmission scans will automatically incorporate the attenuation details of the EEG cap. Thus, final reconstructed images from PET only scanners do not show any attenuation effect due to the components of the EEG cap. This is, however, not the case in hybrid PET-MR scanners, where acquisition of transmission images is not possible. In the hybrid BrainPET-MR scanner, the attenuation correction is performed by using an MR based attenuation correction method [25]. To our knowledge, an explorative study investigating and quantifying the effect of the components of an MR-compatible EEG cap during a simultaneous trimodal study on PET images has not yet been performed.

The MR compatible EEG cap, used during trimodal studies for detecting brain activity signals from the scalp, contains tiny silver/silver chloride (Ag-AgCl) metal electrodes embedded in a plastic housing made of polycarbonate and acrylate. The 511 keV photons emitted by the PET radiotracer may be attenuated by the tiny metal electrodes, resistors, cables and the plastic housing in the EEG cap, leading to possible artefacts in the PET images. A previous study [26] relating to the impact of metal artefacts due to EEG electrodes in brain positron emission tomography **/** computed tomography (PET/CT) imaging, reported that EEG electrodes gave rise to local hot spots and to a significant quantification bias in the PET/CT brain images. These errors, however, did not lead to a modification of the visual interpretation of the brain images or the subsequent diagnoses. However, it should be taken into account that EEG Genuine Grass Precious Gold Disc Electrodes with a disc diameter of 10 mm, supplied by Astro-Med, Inc. (West Warwick, RI, USA), were used in this study, and such disc electrodes are not used in our trimodal studies [2,3]. Furthermore, the attenuation correction was based on CT images and is more sensitive to attenuating material in the PET/CT field of view (FOV). Hence, the study results obtained from PET/CT scanners with a different type of EEG electrodes are not applicable to trimodal PET-MR-EEG. The study presented here, however, is designed to investigate and quantify the influence of EEG caps on PET images measured using hybrid PET-MR scanners, for which the attenuation maps do not include any attenuation information related to the EEG cap.

## Materials and method

A MR compatible BrainCap MR EEG cap (EASYCAP GmbH, Herrsching, Germany) was used to investigate the attenuation effect. The components in the 32 channel BrainCap MR EEG cap and the materials these components are made of are shown in Table 1. In addition to the major components shown in Table 1, the BrainCap MR EEG cap also contains chip resistors (~ 2 mm length, ~ 1 mm breadth) and minute soldering (~ 1 mm length) of copper wire connections between chip resistor and electrode and between chip resistor and thin cables.

**Table 1:** BrainCap MR components and materials.

Component	Material
Cap Fabric	Elastan
Electrode housing (~ 15 mm diameter)	Polycarboate, Acrylate
Electrode (sensor, ~ 2 mm diameter)	Ag/AgCl (99.9% pure)
Thin Cables	Pure Copper, Poly(ethylene terephthalate)

**Fig 1:**
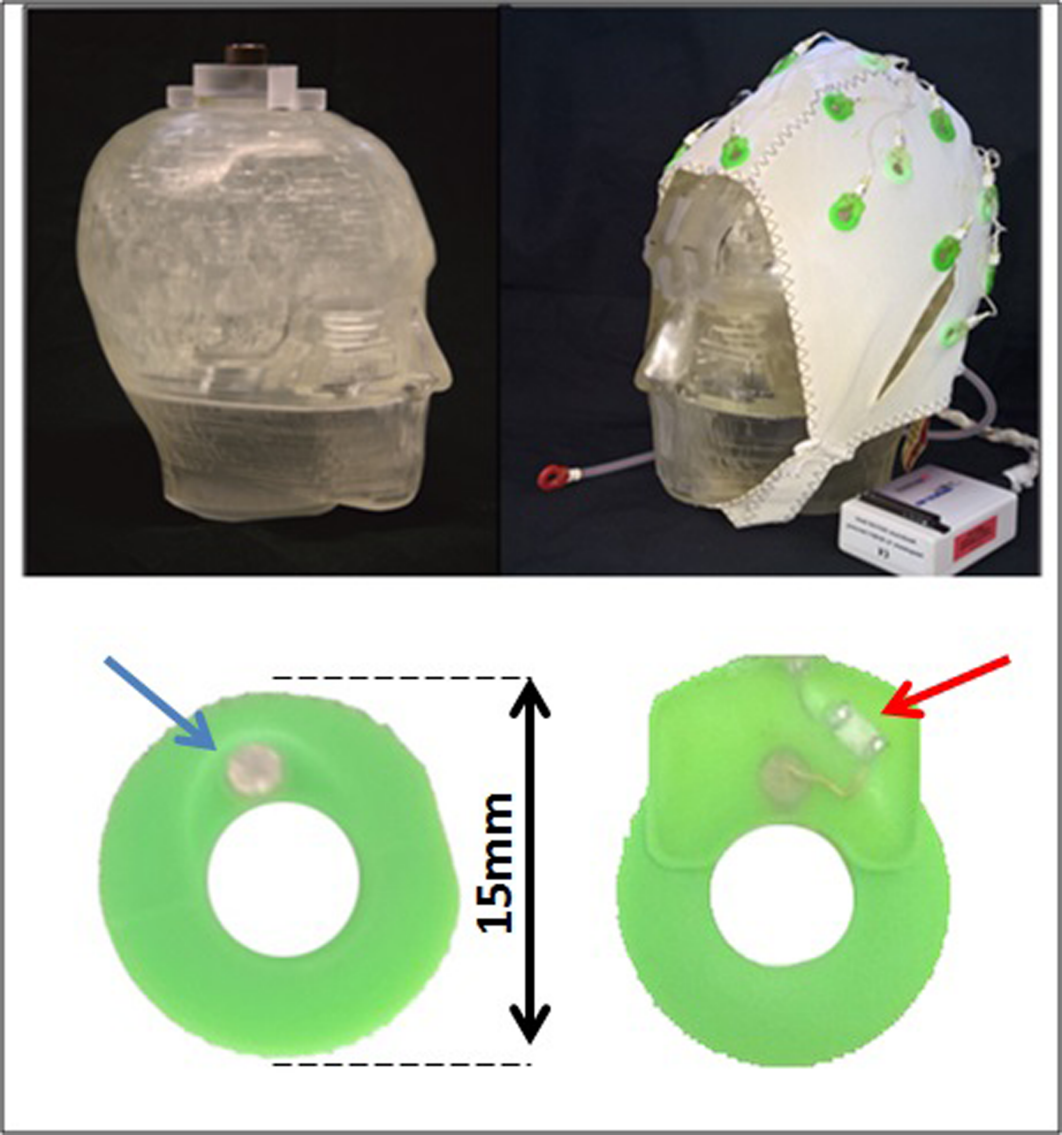
Iida phantom (upper row) without (left) and with (right) MR compatible BrainCap MR EEG cap. The BrainCap MR—Ag/AgCl electrode (indicated by the blue arrow) is embedded in a green plastic housing (lower row—left) and front view of the electrode housing showing the chip resistor (indicated by the red arrow) and soldered wire connection to the Ag/AgCl electrode (lower row—right).

Measurements using the Iida PET phantom [27] (Fig 1) and the BrainCap MR EEG cap were performed with the ECAT EXACT HR+ scanner (*CTI/Siemens*, Knoxville, TN, USA; 32 rings, axial field of view 15.5 cm [28]) and with the 3T-BrainPET-MR scanner (Siemens, Erlangen, Germany; 72 rings, axial field of view 19.2 cm [29,30]).

### Phantom data acquisition and reconstruction

The BrainCap MR EEG cap was placed on the Iida PET phantom (Fig 1) and a 60 minute transmission scan was performed in the ECAT HR+ scanner. The same measurement was repeated without the BrainCap MR EEG cap. The transmission data were iteratively reconstructed (OP-OSEM, 16 subsets, 6 iterations) yielding attenuation maps with and without the EEG cap (AMw and AMwo).

PET emission measurements with simultaneous MR measurements were performed in the 3T-BrainPET-MR scanner on the Iida phantom filled with about 550 ml of radioactive solution (the radioactive solution was prepared by adding 376 MBq [^18^F]Fluorodeoxyglucose ([^18^F]FDG) to one litre water). The PET data of the Iida phantom were also acquired in two sessions, one with the BrainCap MR EEG cap and one without the EEG cap. The data were acquired in list mode and the duration of each session was 30 minutes. The MR protocol incorporated in each session during simultaneous PET-MR measurement and their parameter are as follows:

- magnetisation-prepared rapid acquisition gradient-echo (MP-RAGE) sequence (T1 weighted anatomical imaging sequence, repetition time (TR) = 2250 ms, echo time (TE) = 3.03 ms, field-of-view (FOV) 256 × 256 × 176 mm^3^, voxel size = 1 x 1 x 1 mm^3^, flip angle (FA) = 9°, 176 sagittal slices and a GRAPPA factor of 2 with 70 auto-calibration signal lines)
- ultra short echo time (UTE) sequence (T2* weighted sequence for attenuation correction), TR = 200 ms, TE_1_ = 0.07 ms, TE_2_ = 2.46 ms, FOV = 320 x 320 x 320 mm^3^, voxel size = 1.67 x 1.67 x 1.67 mm^3^, FA = 15°)

The image data were corrected for random coincidences, scatter coincidences, attenuation, and dead time prior to reconstruction. The frame length for the reconstruction of each session was estimated in such a way that the total number of prompt counts within the chosen frame length is equal for both sessions. This was done in order to eliminate the effect of change in radioactivity level between sessions due to Fluorine-18 (^18^F) physical decay. Since FDG in the phantom does not undergo any metabolic changes, such a correction approach is feasible. The estimated frame lengths are 1095 s for the first session with EEG cap and 1300 s for the second session without EEG cap. The start offset was 100 s for both sessions. Reconstruction was done using the OP-OSEM reconstruction algorithm provided by the manufacturer (2 subsets, 32 iterations); finally, a 3D Gaussian filtering with 3 mm kernel was performed. The attenuation map AMwo provided the attenuation correction for both sessions’ data, simulating the real situation of human subject scans, for which attenuation maps of EEG caps cannot be produced in routine study protocols. The acquisition and application of an individual attenuation mask for each volunteer would require additional measurement time in the PET-MR scanner. This would interfere with the optimal timing for bolus infusion in PET-MR protocols and the application of radiotracers [31], thus increasing the radiation exposure inadequately. In addition, due to head motion creating mutual small registration mismatches, an individual mask would not guarantee a more precise PET measurement. Thus, the PET emission images were reconstructed using AMwo. The exact position of AMwo for the BrainPET images was obtained by matching the AMwo to the anatomical MR images of the Iida phantom, obtained during the simultaneous PET measurement.

### Human data acquisition and reconstruction

A similar experiment with two sessions was performed on eight healthy human subjects (age: 28 ± 3.6 years) injected with 202.8 ± 35.1 MBq of [^18^F]FDG at the start of the trimodal experiment and while the subjects were lying inside the BrainPET-MR scanner. The first dynamic emission scan with an EEG cap was started simultaneously with the injection of [^18^F]FDG for a duration of 60 minutes (measurement with cap—MwC); a predefined MR protocol, adapted for measurement with EEG cap, started at the same time. The sequences incorporated in the MR protocol and their parameters are as follows:

- MP-RAGE (same parameters as the mesurement done on the phantom)
- UTE (same parameters as the mesurement done on the phantom)
- spectroscopy (Point Resolved Spectroscopy (PRESS) sequence, TR = 2500 ms, TE_1_ = 14 ms, TE_2_ = 105 ms, number of averages (NA) = 128, voxel size = 25 × 25 × 25 mm^3^, RF pulse centred at 2.4 ppm, 16 step phase cycling)
- echo planar imaging (T2*-weighted EPI sequence, TR = 2200 ms, TE = 30 ms, FOV = 200 x 200 x 108 mm^3^, voxel size = 3.125 x 3.125 x 3.0 mm^3^, FA = 80°, number of slices = 36, number of volumes = 165, eyes closed resting state measurement)

EEG data were also recorded during this simultaneous measurement. After MwC, the subject was taken out from the BrainPET-MR scanner and the EEG cap was removed. After an interval of 17:08 ± 3:56 minutes from the end of MwC, a second emission scan over 20 minutes without the EEG cap was performed (measurement without cap—MwoC) with a predefined MR protocol. The MR protocol during MwoC comprised diffusion tensor imaging (DTI) using a standard double-refocused spin-echo EPI sequence with bipolar gradient pulses (TR = 9100 ms, TE = 87 ms, FOV = 240 x 240 x 136 mm^3^, voxel-size = 1.9 × 1.9 × 1.9 mm^3^, flip angle = 90°, *b*-values = 0, 1000 s mm^-2^, number of slices = 72, number of field gradient directions = 30, NA = 4). The protocols during MwC and MwoC did not include any task. The measurements on the human subjects were part of an ongoing study approved by the Ethics Committee of the Medical Faculty of the RWTH Aachen University, Germany. No additional activity was injected for the purpose of the report presented here. Written informed consent was obtained from all subjects and the study was conducted according to the principles expressed in the Declaration of Helsinki.

The human PET emission data were reconstructed with the same protocol as used for the phantom data (OP-OSEM reconstruction provided by the manufacturer with 2 subsets and 32 iterations). The count rate reduction in PET caused by MR sequences such as EPI and DTI sequences [14] can be, and was indeed corrected by interpolation. The attenuation corrections were performed with template-based attenuation maps [25], which do not contain any attenuation information from the EEG cap. The template-based attenuation map was obtained from a template attenuation map with a corresponding MR template. The former was built as an average image from eight available transmission images performed with the ECAT Exact HR+ scanner (reconstruction: OP-OSEM, 16 subsets, 6 iterations). The MR template was generated with the corresponding and co-registered MR images. The idea of the template-based attenuation correction is based upon the possibility of adapting the MR template to the patient MR image with nonlinear registration (SPM8; Wellcome Department of Neurology, London, UK). The resulting nonlinear transformations are applied to the template attenuation map finally adapting it to the PET data of the subject investigated. Further processing steps were done using the PMOD software package (version 3.4, Zurich, Switzerland), the MPI tool (version 6.48, Advanced Tomo Vision, Kerpen, Germany), the Matlab software package (version 8.5 (R2015a)) and the SPM tool box (version 12).

### Phantom data pre-processing

In order to evaluate the attenuation caused by the electrodes of the EEG cap on the ECAT HR+ attenuation image, the relative difference image in percentage was calculated between AMw and AMwo using the formula:

$$\frac{AMw-AMwo}{AMwo}\cdot 100$$ (1)

The attenuation assessment was done by defining six different VOIs at the position of electrodes on the relative difference attenuation image in percentage. The VOIs were drawn using the tools available in the PMOD software package; the shape and size of the VOIs were drawn to exactly match the attenuation spots on the difference image.

The reconstructed and smoothed FDG-PET Iida phantom image without BrainCap MR EEG cap (PIwoC) was rigidly matched to the FDG-PET Iida phantom image with BrainCap MR EEG cap (PIwC) using the PMOD software package. In order to reveal qualitative differences between PIwoC and PIwC, two professionally experienced physicians were asked to compare the reconstructed emission images of the Iida phantom; the comparisons in each category were blinded.

In order to be able to compare the two images for quantitative assessment, PIwoC and PIwC were normalised to Z-score images relative to the global mean and standard deviation. A relative difference image between Z-score normalised PIwoC and PIwc in percentage was created using the formula:

$$\frac{PIwC-PIwoC}{PIwoC}\cdot 100$$ (2)

A whole phantom binary mask, covering only the grey matter (GM) area in the phantom, was created by thresholding PIwoC using the SPM software package for further processing and calculations. Eight other spherical volumes of interest (VOI) of 9 mm radius were created using the FSL software package. These eight VOIs were directly positioned below the electrodes; the position of the electrodes was determined by rigidly matching the transmission scan images to the phantom emission images. In order to further restrict the calculations to the GM regions of the phantom, only the VOI voxels within the GM region were considered (GM correction). This was achieved by using the whole phantom binary GM mask created previously. In order to quantify the attenuation effect of the components of the EEG cap, the statistical parameters such as mean, median, standard deviation (SD) and interquartile range (IQR) were calculated from the relative difference image in percentage for the whole phantom but only in relation to the grey matter region and for eight other spherical GM corrected VOIs created before. The statistical parameters were calculated using the Matlab software package.

### FDG-PET human data pre-processing

FDG-PET emission data acquired during MwC were reconstructed into 18 different time frames and an average over the last four frames, with a frame length of 5 minutes each—corresponding to 40–60 minutes after [^18^F]FDG injection, was considered for further analysis (FDG-PET image with cap—IwC). Similarly, FDG-PET emission data acquired during MwoC were reconstructed into 4 frames with a frame length of 5 minutes each and averaged into one image (FDG-PET image without cap—IwoC). Before averaging, both IwC and IwoC were smoothed with a 3 mm Gaussian filter and motion corrected; IwoC was decay corrected back to IwC scan time and both normalised to standard uptake value (SUV) images, with respect to body weight and total injected activity of the individual subject. Since FDG uptake is very high in the grey matter of the brain, further processing and computations are done only on the grey matter regions. In order to accomplish this, individual grey matter masks (GM mask) of each subject were created from the IwC co-registered MR structural image (MPRAGE) using the brain extraction tool (BET) [32]; the extracted GM mask then was binarised at a threshold of 10%.

The IwoC was rigidly matched to IwC using PMOD software package and the relative difference image in percentage between IwC and IwoC was calculated using the formula:

$$\frac{IwC-IwoC}{IwoC}\cdot 100$$ (3)

**Fig 2:**
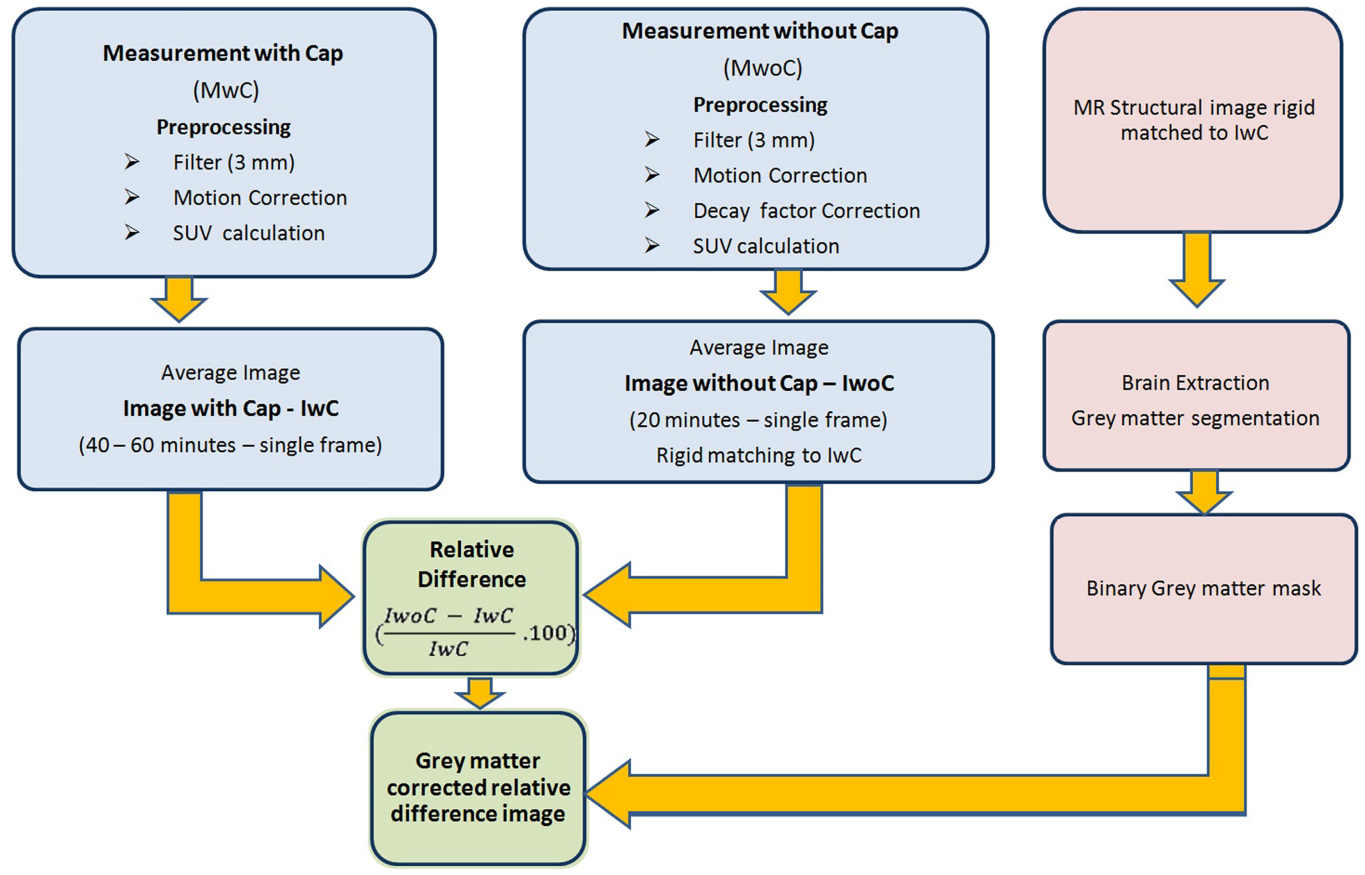
Flow chart showing preprocessing steps for FDG PET human data.

The relative difference image in percentage was corrected for grey matter using the binarised GM mask created before. The processing steps are illustrated in Fig 2.

## Results

FDG-PET phantom and human subject data were pre-processed as explained in the methods section and the results for phantom and human subject data are presented below accordingly.

### Phantom results

**Fig 3:**
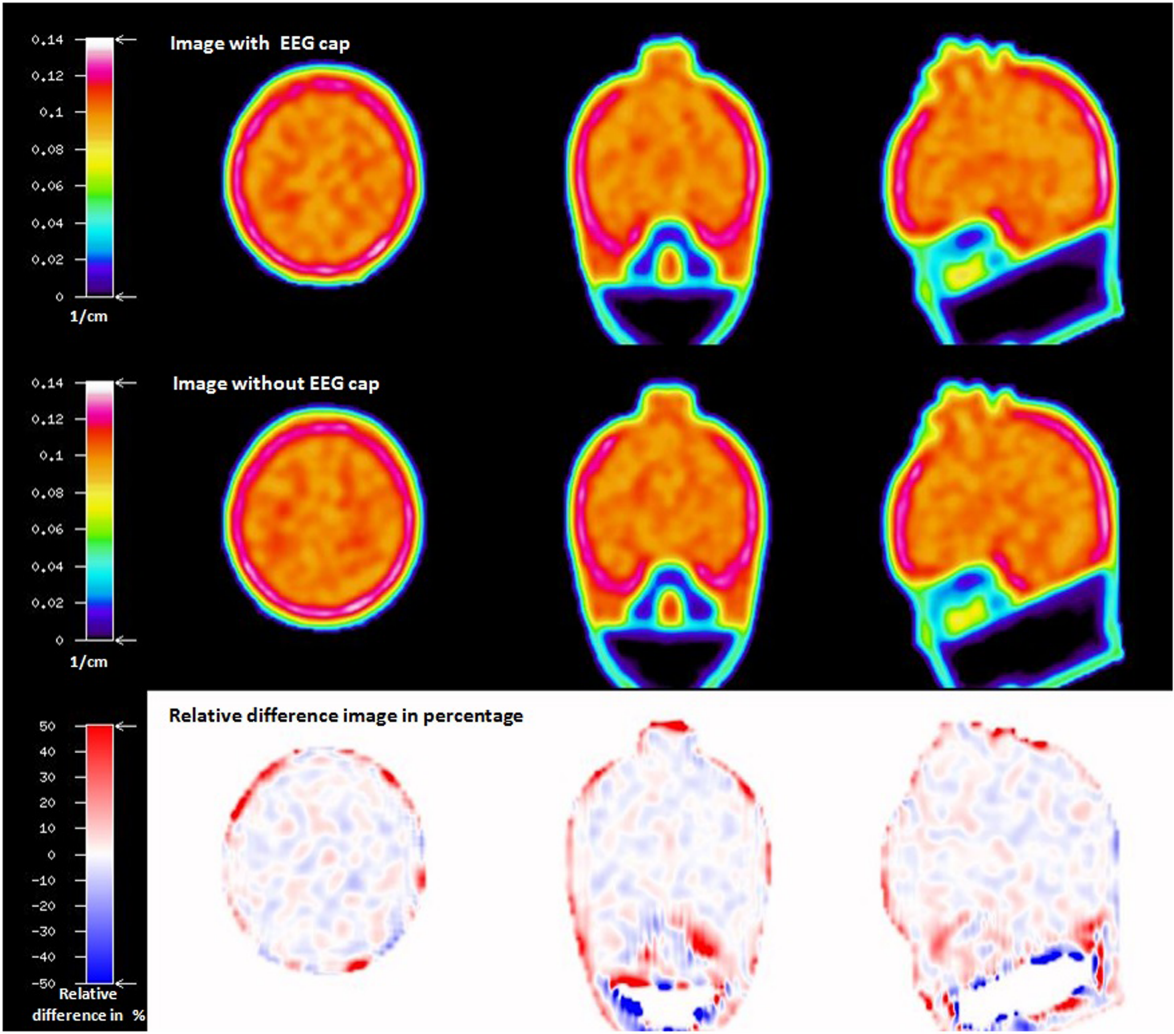
Attenuation maps of the Iida phantom with EEG cap (AMw, upper row) and without EEG cap (AMwo, middle row); relative difference image in percentage (bottom row).

The ECAT HR+ relative difference attenuation image in percentage between AMw and AMwo of the Iida phantom is shown in Fig 3. The attenuation assessment at six different VOIs showed a group average relative difference of about 41.6 ± 24.5% directly on the accessed electrode positions.

**Fig 4:**
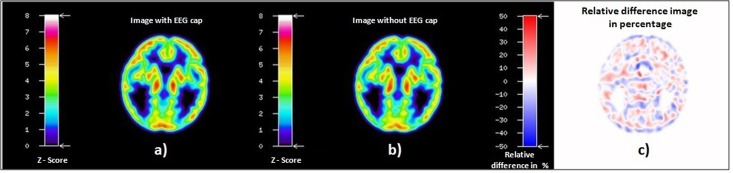
Emission images of the Iida phantom with EEG cap (a), and without EEG cap (b), both reconstructed using the attenuation map without EEG cap; relative difference image in percentage (c).

When comparing the reconstructed emission images of the Iida phantom measured in the 3T-BrainPET-MR scanner, the professionally experienced physicians could not detect any differences between the emission images obtained with and without the EEG cap. In order to underpin this, a relative difference image in percentage between the emission images of the Iida phantom with and without an EEG cap was created (Fig 4) and visually inspected. The difference image showed no artefacts due to the presence of the EEG cap components.

**Fig 5:**
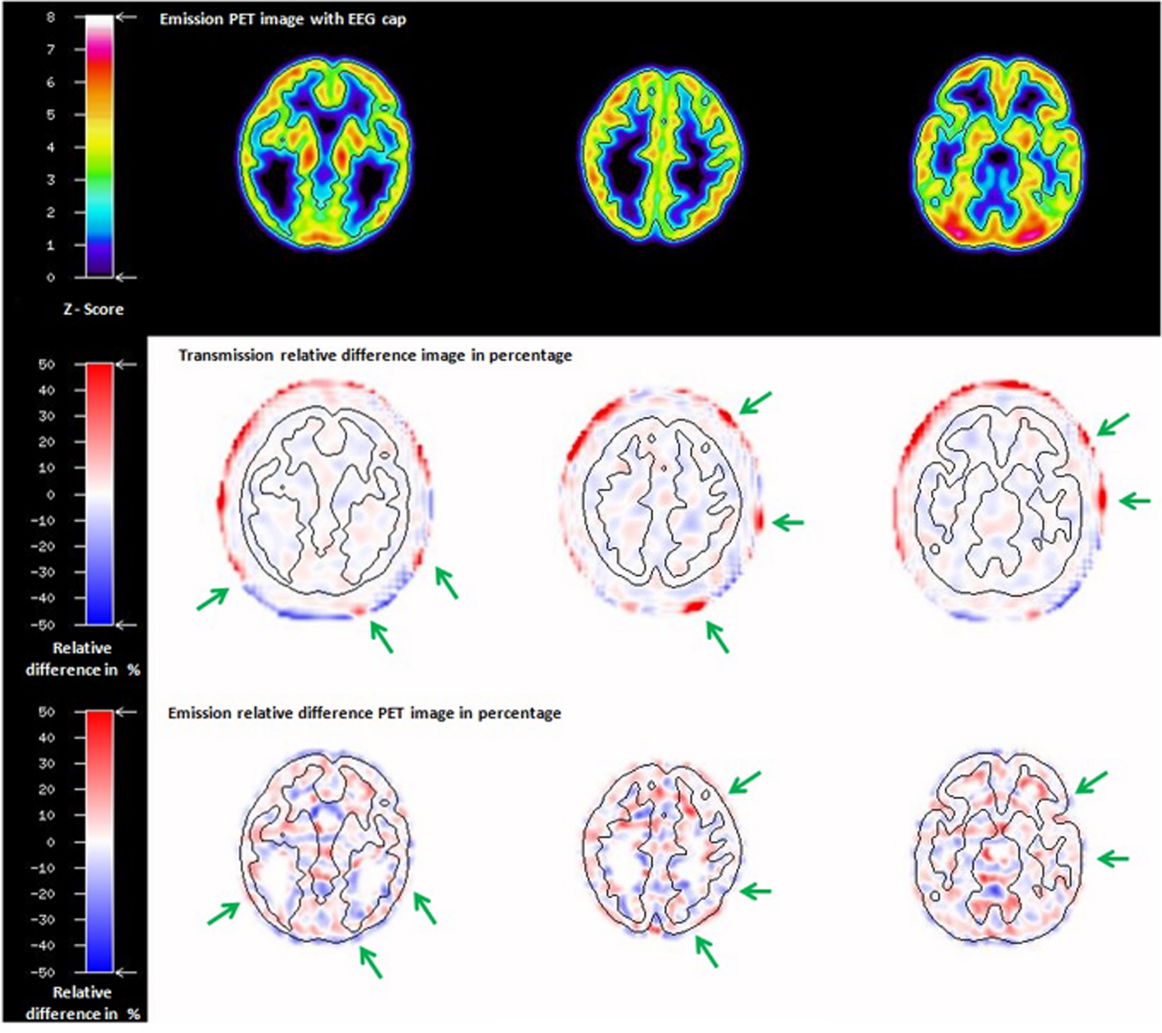
Axial slices at three different axial positions of the emission image of the Iida phantom with EEG cap (upper row), corresponding axial slices of the relative difference images from Fig 3 (transmission image, middle row), and from Fig 4 (emission image, bottom row). The green arrows indicate the position of electrodes in transmission and emission relative difference images in percentage. For visualisation of the position of the ‘brain’ region of the Iida phantom in relation to the electrodes, a black contour corresponding to the outer contour of the emission image is shown on the difference images.

In order to gain a visual impression of the position of the electrodes in relation to the PET emission images of the Iida phantom, the PET emission images were co-registered to the transmission image and all the slices were visually inspected for mutual artefacts due to EEG cap components. Fig 5 shows three different axial slices from the emission image of the Iida phantom with EEG cap, together with the corresponding axial slices of transmission and emission relative difference images in percentage.

**Fig 6:**
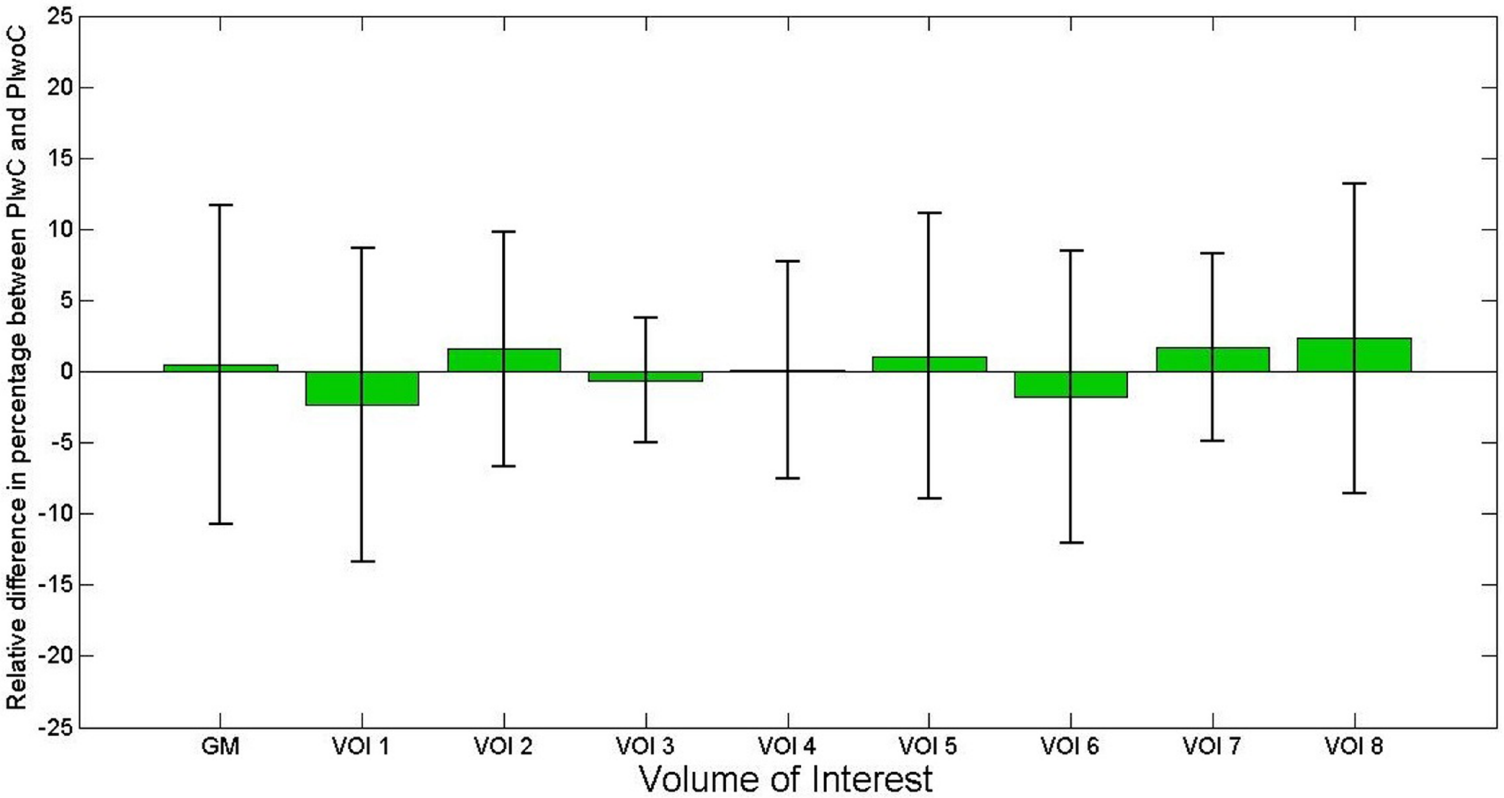
Bar plot showing the mean (green bar) and standard deviation (black lines) of the relative difference emission image of the phantom for whole phantom grey matter and eight spherical VOIs under the position of the electrodes.

The statistical parameters were calculated from the relative difference image in percentage for the whole phantom (only grey matter region) and for the eight spherical VOIs created previously; they are summarised in S1 Table and a visual representation is shown in the bar plot (Fig 6).

### FDG—PET human data results

**Fig 7:**
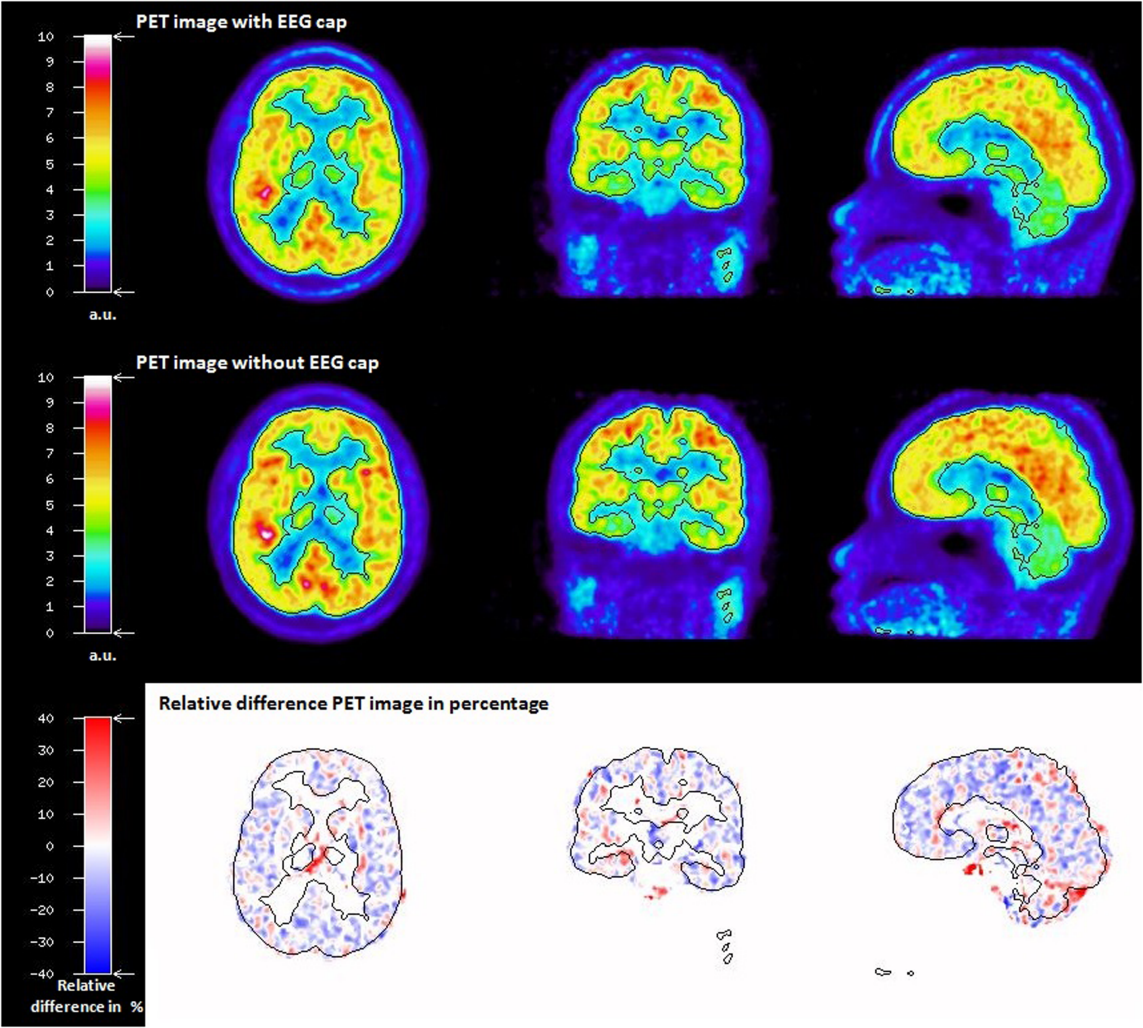
FDG PET SUV image of a human subject with EEG cap (top row) and without EEG cap (middle row), both reconstructed with an AC map without EEG cap; relative difference image in percentage between the above FDG PET images (bottom row).

As stated for the phantom data, the visual inspection of the relative difference image in percentage between IwC and IwoC (both reconstructed with AMwo) of the human subjects also did not show any visible artefacts due to the EEG cap components. Emission PET images with and without EEG cap and the relative difference image of a representative subject is shown in Fig 7. Relative difference images in percentage of the remaining seven subjects are shown in S1 Fig. The statistical parameters calculated from human emission relative difference images in percentage are given in the S2 Table. The histogram plot of the voxel values in the human relative difference images is shown in S2 Fig.

## Discussion and conclusions

The transmission scan performed with the ECAT HR+ scanner on the Iida phantom with the EEG cap clearly shows attenuation on the surface of the phantom scalp caused by the components of EEG cap (Fig 3). The presence of the tiny metal electrodes and chip resistors, plastic housing and other minute soldering materials causing attenuation, generate a higher attenuation correction factor (ACF) for the affected line of response (LOR), consequently leading to an overcorrection along these LORs. The result is a possible overcorrection in the areas directly below the electrodes. In contrast, Figs 4 and 5 clearly illustrate that PET emission images of the phantom showed no significantly visible artefacts due to the presence of EEG cap. The quantitative assessment on the relative difference in percentage of the phantom PET emission images showed the mean values of 0.45(±11.21) % and maximum of -2.36 (±11.04) % on the whole phantom grey matter region and over the eight spherical VOIs, respectively. However, it cannot be concluded that the relative differences shown are only due to the presence of EEG cap. Other factors contributing to this relative difference may be the influence of count rate, statistical uncertainties arising from both the measurement and the reconstruction of PET images, and mismatches due to the rigid matching approach. Furthermore, the emission phantom measurement with and without EEG cap was performed at two different sessions. The counts recorded in a single detector element during two different PET measurements with identical measurement time and phantom radioactivity differ due the Poisson noise of the radioactive events. This noise propagates via the image reconstruction into single image pixels. Therefore, even if the mean values in a VOI are exactly zero between two PET studies, the standard deviation (SD) (and interquartile range (IQR)) will have relatively large values, as tabulated in S1 Table. Only a PET measurement carried out over a very long time, with its better count statistics and optimum reconstruction parameters will result in low SD. Thus, we conclude that even though there might be small artefacts due to the presence of the EEG cap in the emission images, these artefacts do not stand out from the general noise, which includes the factors discussed above. Furthermore, the results from the emission scans of the human subjects show that these additional attenuation effects, caused by the presence of the EEG electrodes, do not lead to any significant visible artefacts. Quantitative assessment on the relative difference images of the human subject data was not performed due to the fact that the metabolism of the FDG tracer changes during the acquisition time. It is well known and illustrated via the whole brain grey matter activity curve, shown in S3 Fig, that FDG accumulates in grey matter over time. Consequently, applying Eq 3 would result in images showing a negative activity distribution. These relative difference images can be observed in Fig 7 and S1 Fig. Even though a quantitative assessment of the human subject brain at different scan times is performed, as done for the phantom emission data, it is not possible to interpret this result because the difference image will show significant changes due to metabolic activity and accumulation of FDG. Factors, such as metabolic activity of the human brain, accumulation of FDG over time, and similar factors, as discussed for the phantom emission measurement in the above paragraph, contributed to the negative relative differences and the large standard deviation values tabulated in S2 Table.

In summary, there may be several factors responsible for the fact that the emission scan images of phantom and human subjects obtained in the PET-MR scanner using an EEG cap did not show any observable variations:

- The EEG electrodes are housed in plastic made of polycarbonate and acrylate, which has attenuation coefficients similar to water (attenuation coefficient calculation is shown in S1 Appendix)—and thus brain tissue.
- The attenuation factor along a line of response, which crosses an EEG electrode, results from the integral through all attenuating material along this line. The attenuation caused by the brain tissue and the bone along this line is much higher than that caused by the EEG electrodes, so their contribution can be regarded as negligible.
- The diameter of the Ag/AgCl EEG electrode is 2 mm and the thickness varies between 1.1 and 1.4 mm. The total area of the 32 Ag/AgCl electrodes only covers about 0.1% of the total area of the head surface (circumference of average human head was considered as 58 cm [33] for this computation. Calculation of percentage of area covered by the electrodes on the surface of the human head is shown in S1 Appendix) so that the percentage of annihilation photons hitting the EEG electrodes on their path from the brain to the PET detectors crystals is very low. Thus, the influence of the lines of responses being attenuated by the EEG electrodes and used as input for image reconstruction can be regarded as negligible.
- The electrode cables used in the EEG cap are very thin and most of the cable bundle is placed out of the field of view of PET detectors. Since the transmission study did not reveal any effect due to the cables, it follows that no noticeable effect should be expected in the emission scan.

In summary, we conclude that the attenuation due to EEG caps at the location of the electrodes is visible and quantifiable in ECAT HR+ transmission scan data; however, its influence is not visible in the reconstructed emission scans from BrainPET-MR scanner. The effects are inconsequential, given that the EEG cap components, such as plastic electrode housing and fabric material, have very low attenuation coefficients and the metal electrodes and chip resistors used are extremely thin.

## Limitations

The results presented in this study are based on a 32 channel BrainCap MR EEG cap, however it may be of interest, especially with the background of source localisation, to study and compare the influence of a larger number of electrodes (64 or 128 channels) on PET emission data. Furthermore, the PET emission images of the human subjects were reconstructed without attenuation information about the EEG cap. The present ethical and radiation protection approvals do not include additional CT scans for healthy human subjects in order to create corresponding attenuation maps. Future studies will be planned and performed to investigate any mutual quantification bias with the attenuation maps containing the electrode information.

## Supporting information

S1 Fig FDG PET relative difference images in percentage between SUV images of human subjects with and without EEG cap. The subjects 2 to 8 are shown.
(TIF)

S2 Fig Histogram plots of the human emission relative difference images in percentage (only the whole grey matter region is considered in the plots.) (TIF)

S3 Fig Activity curves of all 8 subjects showing SUV in whole brain grey matter. (TIF)

S1 Table Statistical parameters calculated from VOIs drawn on the phantom emission relative difference image. (DOCX)

S2 Table Statistical parameters calculated from the whole grey matter region of the human emission relative difference images. (DOCX)

S1 Appendix Calculation of attenuation coefficient for polycarbonate and acrylate and calculation of percentage of area covered by the electrodes on the surface of the human head. (PDF)
