# Supplementary figures and images for: Simultaneous trimodal PET-MR-EEG imaging: Do EEG caps generate artefacts in PET images?
Source: PLoS One. 2017 Sep 13;12(9):e0184743. doi: 10.1371/journal.pone.0184743 (PMC5597218; doi:10.1371/journal.pone.0184743)

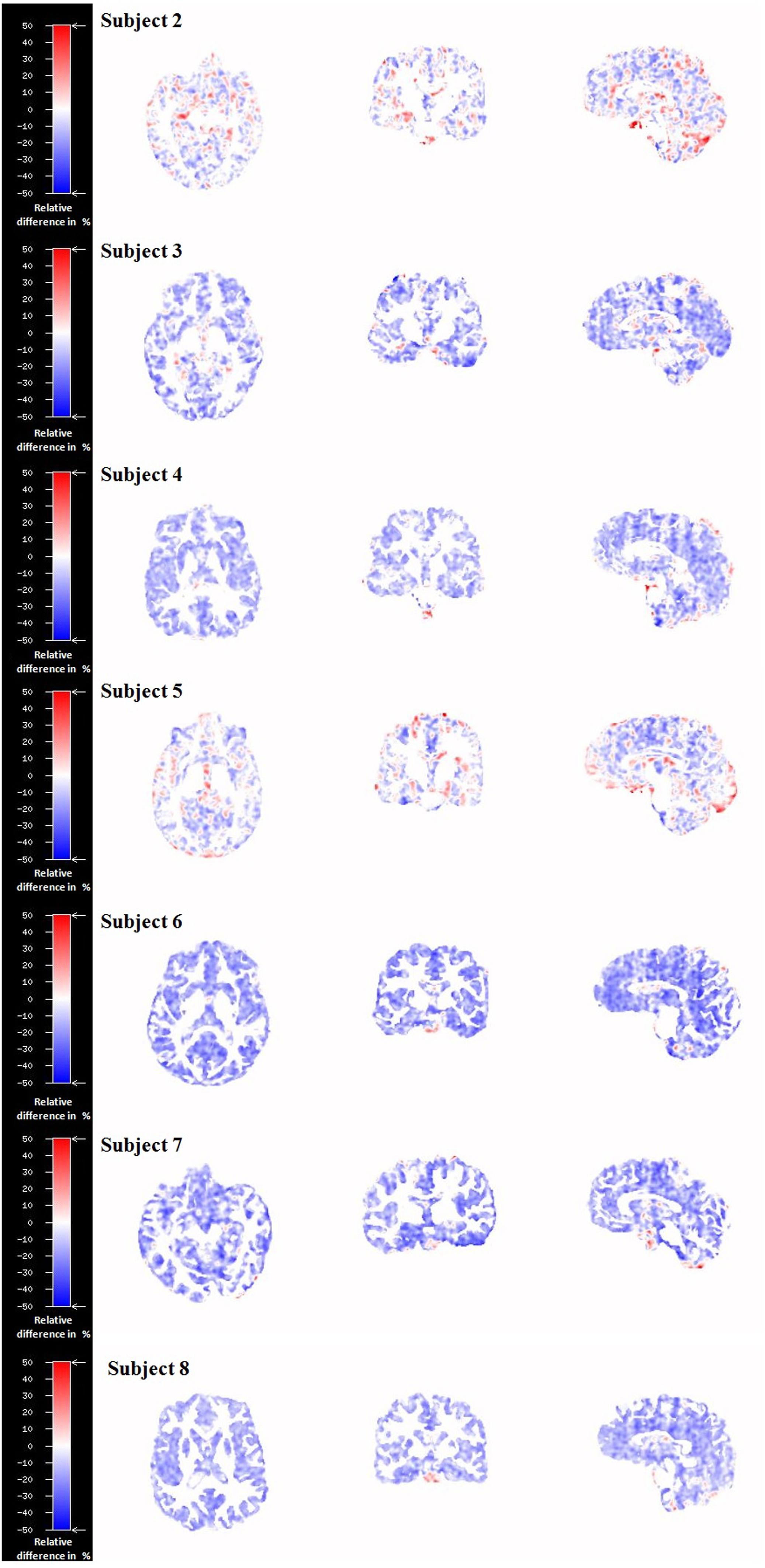

Supplement: S1 Fig — The subjects 2 to 8 are shown. (TIF) [file pone.0184743.s001.tif]

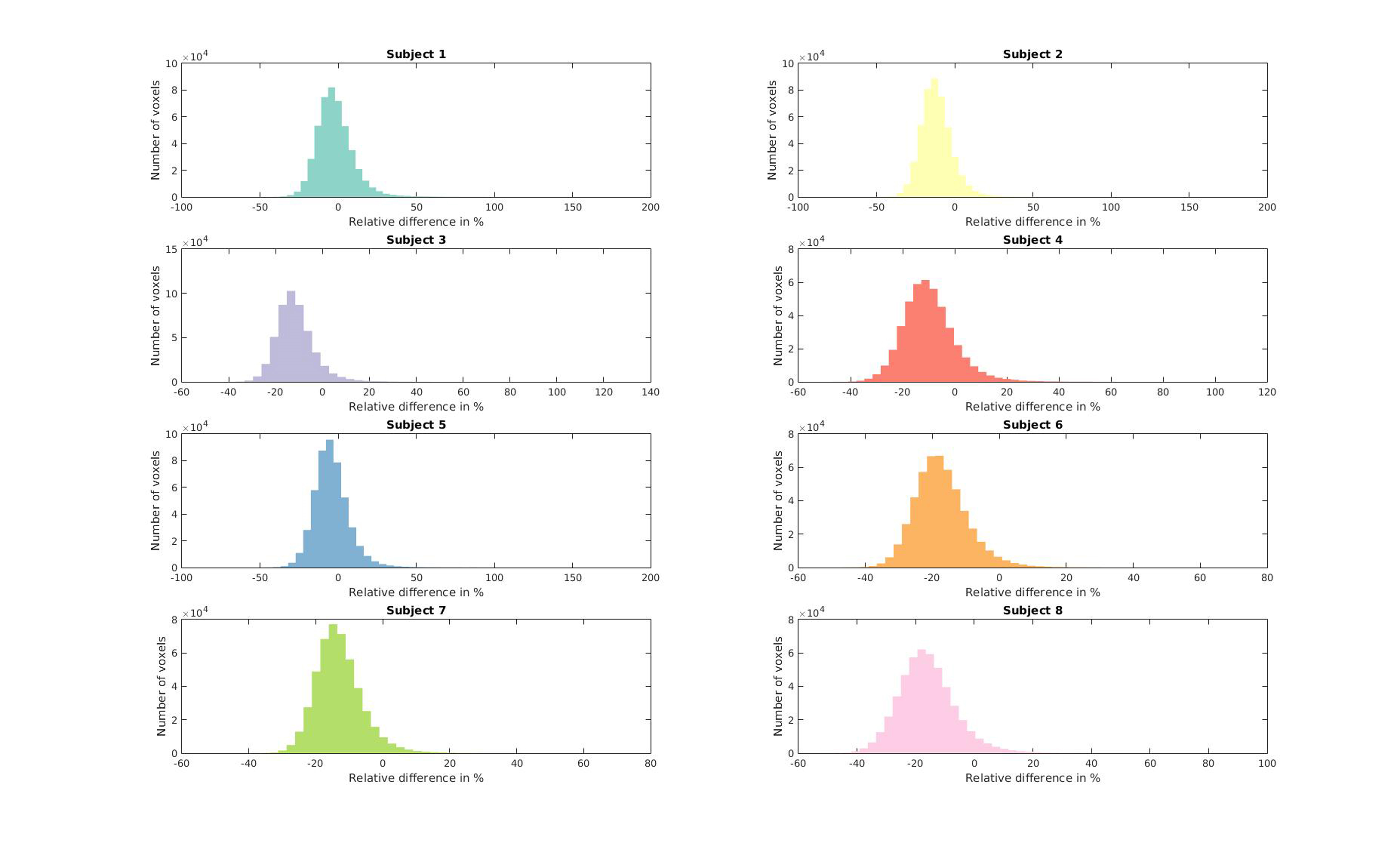

Supplement: S2 Fig — (TIF) [file pone.0184743.s002.tif]

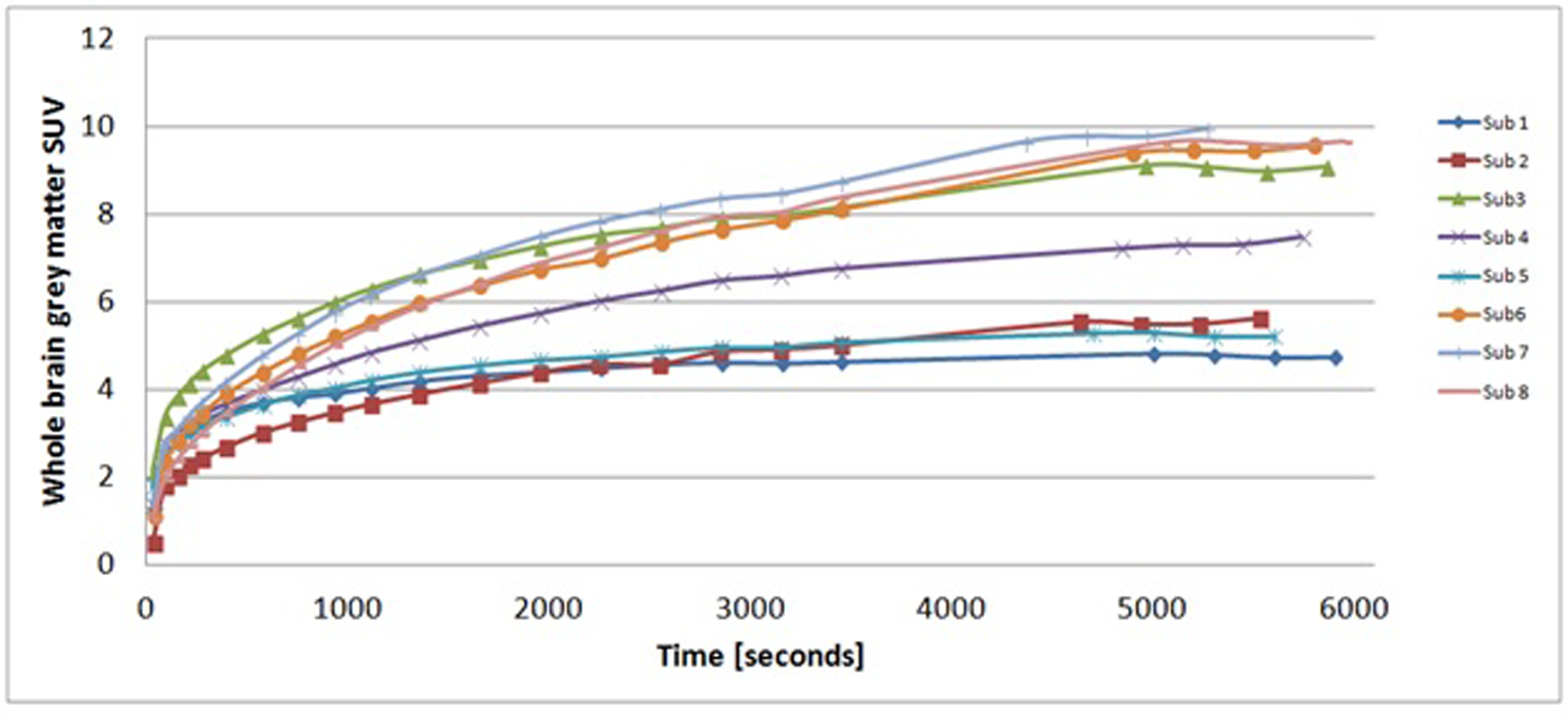

Supplement: S3 Fig — (TIF) [file pone.0184743.s003.tif]
