# Supplementary material for: Simultaneous trimodal PET-MR-EEG imaging: Do EEG caps generate artefacts in PET images?
Source: PLoS One. 2017 Sep 13;12(9):e0184743. doi: 10.1371/journal.pone.0184743 (PMC5597218; doi:10.1371/journal.pone.0184743)
Supplement: S1 Table — (DOCX) [file pone.0184743.s004.docx]

S1 Table: Statistical parameters calculated from VOIs drawn on the phantom emission relative difference image

| **VOI** | **Mean**  **(%)** | **SD**  **(%)** | **Median**  **(%)** | **IQR**  **(%)** |
| --- | --- | --- | --- | --- |
| Whole Phantom  Grey matter | 0.45 | 11.21 | 0.29 | 12.70 |
| VOI 1 | -2.36 | 11.04 | -2.12 | 16.02 |
| VOI 2 | 1.56 | 8.26 | 1.12 | 12.30 |
| VOI 3 | -0.63 | 4.40 | -0.44 | 5.28 |
| VOI 4 | 0.10 | 7.63 | 1.26 | 10.71 |
| VOI 5 | 1.07 | 10.00 | -0.96 | 10.73 |
| VOI 6 | -1.76 | 10.26 | -2.85 | 14.75 |
| VOI 7 | 1.70 | 6.55 | 0.82 | 8.19 |
| VOI 8 | 2.32 | 10.85 | 2.63 | 14.08 |
