# Supplementary material for: Simultaneous trimodal PET-MR-EEG imaging: Do EEG caps generate artefacts in PET images?
Source: PLoS One. 2017 Sep 13;12(9):e0184743. doi: 10.1371/journal.pone.0184743 (PMC5597218; doi:10.1371/journal.pone.0184743)
Supplement: S2 Table — (DOCX) [file pone.0184743.s005.docx]

S2 Table: Statistical parameters calculated from the whole grey matter region of the human emission relative difference images

| **Subject No** | **Mean**  **(%)** | **SD**  **(%)** | **Median**  **(%)** | **IQR**  **(%)** |
| --- | --- | --- | --- | --- |
| 1 | -1.95 | 12.05 | -3.22 | 13.79 |
| 2 | -11.53 | 10.47 | -12.45 | 12.30 |
| 3 | -11.60 | 8.27 | -12.51 | 9.43 |
| 4 | -9.79 | 10.28 | -10.73 | 12.12 |
| 5 | -4.21 | 11.38 | -5.20 | 13.24 |
| 6 | -17.35 | 8.02 | -18.06 | 9.87 |
| 7 | -13.04 | 7.23 | -13.75 | 8.53 |
| 8 | -15.94 | 10.06 | -16.73 | 12.04 |
