# Supplementary material for: Simultaneous trimodal PET-MR-EEG imaging: Do EEG caps generate artefacts in PET images?
Source: PLoS One. 2017 Sep 13;12(9):e0184743. doi: 10.1371/journal.pone.0184743 (PMC5597218; doi:10.1371/journal.pone.0184743)
Supplement: S1 Appendix — (PDF) [file pone.0184743.s006.pdf]

Please refer http://www.ndt.net/article/v05n01/cesareo/cesareo.htm for linear attenuation coefficient of water

Linear Attenuation coefficient of Polycarbonate: [1]

lexan (polycarbonate: C16H14O3)
density of polycarbonate $= 1.2\frac{g}{cm^3}$
mass attenuation coefficient (with coherent scattering) of polycarbonate $(511keV)= 9.109 \cdot 10^{-2}\frac{cm^2}{g}$
Linear Attenuation coefficient = (mass attenuation coefficient) · (density)
Linear Attenuation coefficient of Polycarbonate $= 9.109 \cdot 10^{-2}\frac{cm^2}{g} \cdot 1.2\frac{g}{cm^3} = 0.1093\frac{1}{cm}$

Linear Attenuation coefficient of Acrylate: [2]

Poly(methyl methacrylate) (PMMA) (plexiglass, acrylic, perspex, lucite) [C5O2H8]
density of PMMA $= 1.19\frac{g}{cm^3}$
mass attenuation coefficient (with coherent scattering) of Acrylate $(511keV)= 9.324 \cdot 10^{-2}\frac{cm^2}{g}$
Linear Attenuation coefficient = (mass attenuation coefficient) · (density)
Linear Attenuation coefficient of Acrylate= $9.324 \cdot 10^{-2}\frac{cm^2}{g} \cdot 1.19\frac{g}{cm^3} = 0.1109\frac{1}{cm}$

Percentage of Area occupied by Electrodes:

For this calculation the average circumference of human head was considered as $58cm$ [Bushby et al.(1992)]. And human head was considered as a perfect sphere for this calculation.
Radius of electrodes $r_{electrode}$ was considered as $1mm$
Circumference of Head, $2\pi r_{head} = 58cm \rightarrow r_{head} = 9.23cm$
Area of Head (Sphere) $= 4\pi r_{head}^2 \rightarrow 1070.57cm^2$
Area of 32 electrodes $= 32 \cdot \pi r_{electrode}^2 \rightarrow 1.00512cm^2$
% of Area occupied by Electrodes on Head $= \frac{Area of 32 electrodes}{Area of Head(Sphere)} \cdot 100$
% of Area occupied by Electrodes on Head $= \frac{1.00512cm^2}{1070.57cm^2} \cdot 100\% \approx 0.1\%$

[1] For this calculation values were obtained from (http://web.eecs.umich.edu/ fessler/irt/irt/ct/xray-mass-atten/compound/lexan and http://physics.nist.gov/PhysRefData/Xcom/html/xcom1.html)

[2] For this calculation values were obtained from (http://web.eecs.umich.edu/ fessler/irt/irt/ct/xray-mass-atten/compound/pmma and http://physics.nist.gov/PhysRefData/Xcom/html/xcom1.html)
